# Challenges for Programmatic Implementation of Oral Cholera Vaccine in India

**DOI:** 10.1093/infdis/jiab465

**Published:** 2021-10-20

**Authors:** Debjit Chakraborty, Suman Kanungo, Ranjan Kumar Nandy, Alok Kumar Deb, Asish Kumar Mukhopadhyay, Shanta Dutta

**Affiliations:** 1 Division of Epidemiology, Indian Council of Medical Research-National Institute for Cholera and Enteric Disease, Kolkata, India; 2 Division of Bacteriology, Indian Council of Medical Research-National Institute for Cholera and Enteric Disease, Kolkata, India

**Keywords:** oral cholera vaccine, India, challenges, program, operation

## Abstract

Cholera remains a major contributor of diarrheal diseases and leads to substantial morbidity and mortality, particularly in low socioeconomic settings. Nonavailability of a national cholera control plan in India, compounded by underreporting of cholera cases and deficient accurate cholera hotspot estimates, has made cholera control a challenge. Obstacles in the programmatic introduction of oral cholera vaccine (OCV) lie within the infrastructure—stockpile, costing, distribution system, cold-chain mechanism, vaccine logistics, and lack of strengthened surveillance systems for adverse events following immunization. Sustained political commitment along with collaboration of people working in the media will also determine the policy outcome of OCV introduction in India.

Cholera remains a major contributor of diarrheal diseases and leads to substantial morbidity and mortality, particularly in low socioeconomic settings without proper management. In a country like India where 70% of the population reside in rural areas, cholera continues to be a menace. Estimates show over 400 million people in India are at risk of cholera, with 675 188 cases and 20 256 deaths occurring annually [1]. Many of India’s worst cholera outbreaks can be traced back to the Ganges delta, including the Bay of Bengal. As cholera spreads through unhygienic, contaminated food and water, the Ganges is a perfect breeding ground. The Ganges delta provides a source of water for millions of people living in India, using contaminated water for bathing and cooking. The tropical climate and poor sanitation and hygiene further compound the problem [2].

Although an accurate estimate of the number of cholera cases in India is presently unavailable, it is evident that cholera outbreaks have been consistently reported over the years from different regions of India. According to the Integrated Disease Surveillance Program, 559 cholera outbreaks were reported in India during 2009 to 2017, Karnataka and West Bengal being the top contributors. Cholera modelling by Ali et al revealed that of 36 states in India, 24 states reported at least 1 cholera outbreak in the last 10 years, and 13 states were cholera endemic [1]. Seventy-eight districts were identified as having multiple cholera outbreaks, notifying at least 1 every year in the last 5 years. Of these 78, 35 districts were known to be nonendemic districts. Consequently, the problem of cholera as a continuous public health concern in India cannot be ignored [1].

The World Health Organization (WHO) established a global task force on cholera control (GTFCC), which aims to eradicate cholera by 2030. While this task force emphasizes safe water, sanitation, and hygienic (WASH) measures, cholera vaccines play an additional role in reducing cholera burden, particularly in overcrowded slums with poor hygiene and environmental conditions.

Two oral cholera vaccines (OCV), namely Shanchol and Euvichol, are modified bivalent killed whole-cell–based vaccines, targeted at adults and children older than 1 year. WHO recommends administration of 2 doses at an interval of 2–6 weeks, with a booster dose at 3 years, to all eligible age groups in high-risk and vulnerable areas, particularly in a limited-resource scenario [3].

Several vaccine trials conducted by investigators from the National Institute of Cholera and Enteric Diseases under the Indian Council of Medical Research (ICMR-NICED) demonstrated a protective efficacy of ≥65%, almost consistent from 2–5 years postvaccination in the case of whole-cell killed OCV [4, 5]. Thus, evidence generated from the Indian population has already provided the foundation for policy translation for the use of OCV for prevention and control of cholera by 2030, in line with GTFCC goal. Despite evidence of successful OCV implementation in cholera-endemic countries like Yemen, Somalia, and Iraq, targeted introduction of OCV into a national program in India is still a distant reality. This study looked into the various challenges in different dimensions that have limited the programmatic implementation of OCV in India.

## METHODS

This article was based on review of available literature in the public domain along with analysis of hospital-based acute diarrheal disease surveillance data at ICMR-NICED, Kolkata, India. The goal of the review was to summarize evidence available so far on various challenges and bottlenecks for OCV implementation in India. The literature search was conducted using PubMed and Google Scholar, with the keywords “India,” “cholera,” “OCV,” “oral cholera vaccine,” “challenges,” “acute watery diarrhoea (or diarrhea),” and “immunization program.” Publications were included if based on Indian data or Indian scenarios and published within the last 10 years (since 2011). Duplicate reporting of data was addressed by removing the same data from different sources. Papers not written in English were excluded. We sought to document the magnitude of underreporting of cholera cases to the WHO by the Central Bureau of Health Intelligence (CBHI) through the publication of the National Health Profile of India since 2005. This exercise was aimed at demonstrating the extent of the data gap on cholera in the current national reporting systems.

## RESULTS

**Figure 1.**
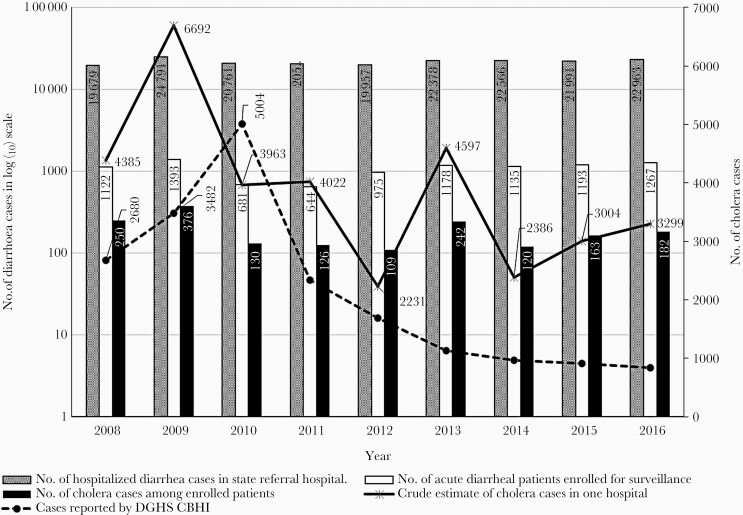
Comparison of crude estimates of culture-confirmed cholera cases admitted per year at one state referral hospital in Kolkata with numbers of cases per year in the national health profile reports of India (reported by Director General of Health Services-Central Bureau of Health Intelligence [DGHS-CBHI]).

Figure 1 clearly demonstrates consistent underreporting of confirmed cholera cases to the Director General of Health Services (DGHS)-CBHI. The data shown were significantly lower (mean difference 1726, SD 1372) than the estimated cholera cases admitted to the state referral hospital (Infectious Disease and Beleghata General Hospital) in Kolkata. We conducted diarrhea surveillance in this hospital and obtained stool samples from admitted diarrhea cases through systematic sampling. Thus, we identified the cholera isolation rate following culture of stool samples from enrolled patients under surveillance. Finally, by multiplying the cholera isolation rate by the total number of admitted diarrhea cases, we derive a crude estimate of cholera cases in this hospital. The national health profile maintained by DGHS-CBHI is a national portal intended to capture all cholera cases reported in India, but we observed the number reported cases in the portal was fewer than the estimated number of cholera cases at this single hospital. Although the reason behind the underreporting is not fully understood, it has certainly reduced the importance of cholera as a disease of public health concern.

Major obstacles in programmatic introduction of OCV lie within the infrastructure—the distribution system, cold-chain mechanism, vaccine logistics, and lack of strengthened surveillance systems for adverse events following immunization to support OCV introduction (Box 1). Because 2 doses of vaccine need to be administered within a minimum of 2 weeks, it requires a supply of vaccine at a large scale, which may not be eligible for free of cost supply by the Global Alliance on Vaccine Initiative (GAVI). Moreover, administration of OCV to subjects aged > 1 year is in discordance with the current universal immunization program in India [6].

Box 1. **Challenges for Programmatic Implementation of Oral Cholera Vaccine in India**

Dilemmas for policymakers

- Absence of laboratory-based surveillance, therefore priority
- Apprehension of negative impact on trade, travel, and commerce
- Hotspots based on seroincidence not available
- National cholera control plan not in place
- Targeted campaign vs mass immunization.

Programmatic challenges

- Two doses to be given with a gap of 2 weeks
- Hotspot regions not identified, consequently a large population unit (district/state) may need to be covered
- Requires supply of vaccine at a large scale
- Administration of oral cholera vaccine (OCV) to subjects aged >1 year is in discordance with the current universal immunization program in India
- Demand identification for vaccine to encourage vaccine manufacturers for uninterrupted supply
- Financial implication—cost vs return on investment.

Operational challenges

- Upgrade infrastructure, including distribution channels
- Increase cold-chain system capacity
- Vaccine storage capacity and logistics mobilization at large scale
- Evaluation of adverse events following immunization surveillance for ability to support OCV introduction
- Accurate vaccine demand forecasting and stock management to minimize vaccine wastage.

Absence of accurate cholera hotspot data limits vaccine demand forecasting and efficient stock management and wastage minimization. In India currently, OCV-specific training is needed but resources are very scarce. Development of a training program, along with communication tools, messaging, and intersectoral coordination, is a daunting task but is vital for efficient rollout of any vaccine. Social mobilization, advocacy, and campaigning activities are also essential to disseminate information to overcome vaccine hesitancy at individual and community level. Innovation in health communications and community mobilization has been recognized as an efficient strategy for successful vaccine implementation, as evidenced by the Pulse Polio Immunization Program in India [7].

Presently, a vaccination program may be most suitable as a response measure to a cholera outbreak. However, tackling issues like waning immunity, migration, and generation of new birth cohorts into a community calls for repetition of vaccination at a specified periodicity as long as needed to control cholera outbreak. In the absence of documented cholera hotspots in India and considering its huge population of around 1.3 billion, the feasibility and cost-effectiveness of vaccination roll out remains a very serious concern for policymakers, particularly in the context of decreasing gross domestic product (GDP) and further diminution of the proportion of GDP spent on public health [8, 9].

These oral vaccines are part of the nonemergency reserve stockpile of GTFCC. The United Nations Children’s Fund (UNICEF) also maintained the stockpile (around 4.3 million OCV doses in 2016), the bulk of which was intended for cholera-endemic countries [10]. Current OCV production capacity, particularly Shanchol which has been licensed in India, is inadequate considering the population of India and its realistic demand.

This uncertainty from the manufacturer of Shanchol, Shantha Biotech, Hyderabad, regarding expansion of OCV production to fulfil the requirement in India is another major impediment for vaccine roll out [11]. A substantial portion of OCV manufactured in the country is already precommitted to the WHO global vaccine stockpile [12]. India is a producer of large quantities of many vaccines, which include donation of vaccines to other countries. The country`s preference may remain in manufacturing indigenous vaccine, which requires a huge enhancement in the country’s vaccine manufacturing capacity.

## DISCUSSION

One of the major challenges in controlling cholera is the nonavailability of a national cholera control plan in India. To prioritize any plan for elimination of a disease, a true estimate of the disease is very important. Gross underreporting of cholera cases has exacerbated the difficulty in obtaining an accurate estimate. Anticipation of a detrimental effect on trade, tourism, and political pressure may also be the cause of underreporting and, therefore, cholera control gradually tends to lose priority for policymakers. It is thought that underreporting may take place at the local, state, and national levels, hiding the actual magnitude of the problem [13]. Despite the presence of the Integrated Disease Surveillance Program in the country for more than 15 years, only cholera outbreaks are reported in India, ignoring the daily occurrence of cholera cases in sporadic infection. In the absence of authentic laboratory surveillance data, determination of an outbreak threshold also becomes subjectively biased. In the absence of adequate laboratory facilities, particularly at peripheral health settings, acute diarrheal diseases cases are mostly managed by empirical antimicrobial therapy, contributing to the threat of emerging antimicrobial resistance.

Because OCV is targeted at children older than 1 year, OCV vaccination needs to be ideally coupled with a universal immunization program, which mostly targets the infant population. This should be recognized as an opportunity as the birth cohorts can be followed for OCV administration. Feasibility studies could be used to explore complementary and alternative strategies to routine vaccination campaigns. Evidence synthesis at local and regional levels and evidence-driven decision making may be effective in the Indian setting where acknowledging the diversities across demography, climate, and disease profile determines the success of any intervention program [14]. A comprehensive multisectoral approach to prevent cholera, which holistically includes interventions to improve water quality, sanitation and hygiene, cholera treatment, and outbreak response, is an integral part of a cholera elimination strategy [15].

The Indian National Technical Advisory Group on Immunization (NTAGI) set up a working group on cholera control in 2016, who meet from time to time to disseminate their recommendations [1, 8]. Sustainability of any vaccination program is the key concern for NTAGI, which needs an uninterrupted supply of vaccines and operation funding [3].

Despite many challenges, there remains a light at the end of the tunnel. On approval from ICMR, ICMR-NICED has initiated a study to estimate the seroincidence of cholera at national and regional levels along with mapping of cholera hotspots. These data may be leveraged during subsequent years for informed public health decision making. Strategic outcomes may be derived from this work, which will facilitate overcoming the existing barriers.

The GTFCC Global Roadmap to End Cholera by 2030 may be implemented in a step wise approaches. This may enable policymakers to facilitate development of a national cholera control plan, which would be aimed at both outbreak response and overall cholera burden reduction. As a part of the plan, targeted OCV implementation may be initiated through a hotspot-based approach and subsequently may be scaled up as suggested by the evidence. Harmonization with WASH programs and multisectoral convergence will be imperative for successful implementation of OCV in all sectors. Collaborations across relevant state government departments (eg, Departments of Health, Environment, Education, and Women and Child Development) would be instrumental in overcoming initial apprehension and vaccine hesitancy, and limitation of logistics problems, and thus will address vaccine preparedness.

Surveillance systems, particularly laboratory-based surveillance, needs to be augmented so that true numbers of cholera cases may be estimated, which would be helpful in assessing the impact of a vaccination program. GTFCC is launching a country support platform (CSP) for cholera in 5 countries, to support and facilitate cholera control activities. Establishment of a CSP in India will certainly be a critical step towards overcoming many challenges.

Lastly, a sustained political commitment at all levels is necessary for successful implementation of an OCV program, which is possible only through evidence-based policy advocacy. Collaboration with people working in the media is also of great importance because communication remains a key determinant in the success of any vaccine implementation program.

## Notes

***Author contributions***. D. C., S. K., and S. D. conceived the study. D. C., S. K., R. K. N., A. K. D., A. K. M., and S. D. performed the literature search and data analysis. D. C., S. K., A. K. D., and S. D. drafted the manuscript. All authors read and approved the final version.

***Potential conflicts of interest.*** All authors: No reported conflicts of interest. All authors have submitted the ICMJE Form for Disclosure of Potential Conflicts of Interest. Conflicts that the editors consider relevant to the content of the manuscript have been disclosed.

Presented in part: 15th Asian Conference on Diarrhoeal Disease and Nutrition, Dhaka, Bangladesh, January 2020.
